# Supplementary material for: Professional decision making with digitalisation of patient contacts in a medical advice setting: a qualitative study of a pilot project with a chat programme in Sweden
Source: BMJ Open. 2022 Jan 16;11(12):e054103. doi: 10.1136/bmjopen-2021-054103 (PMC8640641; doi:10.1136/bmjopen-2021-054103)
Supplement: Supplementary data [file bmjopen-2021-054103supp001.pdf]

Semi Structured Interview template

Information about the study

Who am I/are we that is/are conducting the interview?

The researchers carrying out the study are interested in digitalisation and work environment. The interview will cover different aspects of these topics.

There are no correct or incorrect answers. We want to learn from your experience. The interview will take around 45 minutes to 1 hour

Have you managed to read the information we sent you? If not we read the information.

Is there anything you would like to ask about?

Part 1: Background

Can you tell me a little about who you are? Allow the informant to talk first by him/herself, and then follow up with questions so that the following are covered:

(Gender), age
Can you describe your work situation now?
What is your job/profession?
How long have you been working at 1177?
Where did you work before?

Part 2: The chat

Now we will focus on how you work with the chat. I will ask you what it's like to use the system at work, so when you answer, I would like you to focus on your use of the systems and not on the problems the patients have.

Can you describe what you do when a patient you shall take care of enters the chat?
What systems do you use for chat during your working day?
Are there any differences between them for the user?
What systems do you use for chat most of the time?
What's it like to use Doktrin?
What positive aspects do you encounter using Doktrin?
What negative aspects do you encounter using Doktrin?
How difficult is it to change between Doktrin and the other systems?
What tasks do you handle using the other systems?
Have you had any problems with the other systems?
Do you get any support to solve the problems that arise from using the systems?
Have you needed to call a patient? Why?
Would you like to change the technical equipment that you use the systems on?
Do you ever work anywhere else than here on 1177 when you are on chat duty?
Would you like to work from home?
Does the patient ever come back and tell you how it went?
(To nurses): Do you ever get any feedback from the doctors about whether your case was correctly categorised?
How much cooperation do you have with the doctors/nurses?
Is it something you would like to change in your digital work environment?

Part 3: Telephone

Now we will focus on when you work on the phone and the systems you use there.

Can you describe what you do when a patient you shall take care of contacts you on the telephone and which systems you use?
Is it the same system you use for the chat? Or are there any others?
What systems do you use most of the time for working on the telephone?
How difficult is it to change between the systems on the telephone?
How do you use the systems?
Do you have any problems using the other systems, the ones you don't use in the chat?
Do you get any support to solve the problems that arise from using all of the systems when you work on the phone?
Do you ever work anywhere else than here on 1177 when you are on the telephone?
Is there anything you would like to change in your digital work environment (unless I have asked before)?
What do you think are the advantages of working with the chat?

What are the advantages of working on the telephone?
Which is more stressful? Why?
Which do you prefer? Why?

Part 4: Conclusion

We will now conclude the interview.

Is there anything you want to add or something you want to ask?

If I may sum up ... (summary of previous answers). Is that right?

Thank you very much for allowing me to interview you - it has been very informative and will help us a lot in our research project.
