# Supplementary material for: Professional decision making with digitalisation of patient contacts in a medical advice setting: a qualitative study of a pilot project with a chat programme in Sweden
Source: BMJ Open. 2022 Jan 16;11(12):e054103. doi: 10.1136/bmjopen-2021-054103 (PMC8640641; doi:10.1136/bmjopen-2021-054103)
Supplement: Author's manuscript [file bmjopen-2021-054103.draft_revisions.pdf]

BMJ Open is committed to open peer review. As part of this commitment we make the peer review history of every article we publish publicly available.

When an article is published we post the peer reviewers' comments and the authors' responses online. We also post the versions of the paper that were used during peer review. These are the versions that the peer review comments apply to.

The versions of the paper that follow are the versions that were submitted during the peer review process. They are not the versions of record or the final published versions. They should not be cited or distributed as the published version of this manuscript.

BMJ Open is an open access journal and the full, final, typeset and author-corrected version of record of the manuscript is available on our site with no access controls, subscription charges or pay-per-view fees (http://bmjopen.bmj.com).

If you have any questions on BMJ Open's open peer review process please email info.bmjopen@bmj.com

BMJ Open

Altered Professional Decision Making with Digitalisation of Patient Contacts in a Medical Advice Setting

Journal:	*BMJ Open*
Manuscript ID	bmjopen-2021-054103
Article Type:	Original research
Date Submitted by the Author:	16-Jun-2021
Complete List of Authors:	Cajander, Åsa; Uppsala Universitet Teknisk-naturvetenskapliga fakulteten, Department of Information Technology<br>Hedström , Gustaf ; Uppsala Universitet, Department of Public Health and Caring Sciences<br>Leijon , Sofia ; Uppsala Universitet, Department of Information Technology<br>Larusdottir , Marta; Reykjavik University School of Computer Science, School of Computer Science
Keywords:	Health informatics < BIOTECHNOLOGY & BIOINFORMATICS, Information technology < BIOTECHNOLOGY & BIOINFORMATICS, Telemedicine < BIOTECHNOLOGY & BIOINFORMATICS, Human resource management < HEALTH SERVICES ADMINISTRATION & MANAGEMENT, Organisation of health services < HEALTH SERVICES ADMINISTRATION & MANAGEMENT, Public health < INFECTIOUS DISEASES

SCHOLARONE™
Manuscripts

I, the Submitting Author has the right to grant and does grant on behalf of all authors of the Work (as defined in the below author licence), an exclusive licence and/or a non-exclusive licence for contributions from authors who are: i) UK Crown employees; ii) where BMJ has agreed a CC-BY licence shall apply, and/or iii) in accordance with the terms applicable for US Federal Government officers or employees acting as part of their official duties; on a worldwide, perpetual, irrevocable, royalty-free basis to BMJ Publishing Group Ltd ("BMJ") its licensees and where the relevant Journal is co-owned by BMJ to the co-owners of the Journal, to publish the Work in this journal and any other BMJ products and to exploit all rights, as set out in our licence.

The Submitting Author accepts and understands that any supply made under these terms is made by BMJ to the Submitting Author unless you are acting as an employee on behalf of your employer or a postgraduate student of an affiliated institution which is paying any applicable article publishing charge ("APC") for Open Access articles. Where the Submitting Author wishes to make the Work available on an Open Access basis (and intends to pay the relevant APC), the terms of reuse of such Open Access shall be governed by a Creative Commons licence – details of these licences and which Creative Commons licence will apply to this Work are set out in our licence referred to above.

Other than as permitted in any relevant BMJ Author's Self Archiving Policies, I confirm this Work has not been accepted for publication elsewhere, is not being considered for publication elsewhere and does not duplicate material already published. I confirm all authors consent to publication of this Work and authorise the granting of this licence.

For peer review only

Altered Professional Decision Making with Digitalisation of Patient Contacts in a Medical Advice Setting

Åsa Cajander[1], Gustaf Hedström[2], Sofia Leijon[1], Marta Larusdottir[3]

1.Department of Information Technology, Uppsala University
2.Department of Public Health and Caring Sciences, Uppsala University
3.School of Computer Science, Reykjavik University

Corresponding author:
Åsa Cajander, Uppsala University, Lägerhyddsvägen 2, Box 337, 751 05 Uppsala
Email: asa.cajander@it.uu.se
Phone: 0704-250 786
Fax: --

Key Words: automation, eHealth patient-centric services, decision making, assessment, healthcare professionals

Word count: 3886

Abstract

Objectives: Patient e-services are increasingly launched globally to make health care more efficient and digitalised. One area that is digitalised is medical advice, where patients asynchronous chat with nurses and physicians. However, little is known about the effects on professional decision making and assessment, which are parts of professionalism theories.
Setting: Health care professionals working in a pilot project with a chat program for patients in a medical advice setting.
Participants: This paper is based on contextual inquiries, and 17 interviews with nurses and physicians.
Primary and secondary outcome measures: The analysis was inductive and based on theories of decision making.
Results: The interviews were thematically analysed. Three categories emerged; 1) Decision basis with questions to patients not tailored for their work, 2) Reduced trust in written communication and 3) Reduced opportunity to obtain information through chat communication**.**
Conclusion: The results indicate that asynchronous chat with patients reduces the opportunity for nurses and physicians to obtain and use professional knowledge and discretionary decision-making. Furthermore, the system's design increases uncertainty in assessments and decision-making, which reduces the range of occupational professionalism.

Strengths and limitations of this study

1. The methodological strength of the study is the combination of field studies with an interview study.
2. Another methodological strength is that this case study is based on an implementation project and not a research intervention.
3. A final methodological strength is the unique and highly relevant research question that has not been explored before.
4. The limitation of the study is the number of nurses (N=9) and physicians (N=8) in the interview study. However, all the nurses and physicians joining in the proof of concept project was interviewed.

1. Introduction

The idea that new technology will solve current or future difficulties is not a recent phenomenon. The digitalisation of healthcare is no exception when new technologies such as AI have been highlighted as solutions to existing problems [1,2]. However, follow-ups have shown that the systems rarely deliver the expected benefits regarding, among other things, increased efficiency and safety [3–6]. Moreover, the results from eHealth implementations vary depending on the solution and organisational context [7].

One recent area with varying implementation results is asynchronous communication platforms where health care professionals and patients interact [8–12]. For example, Jhala and Menon [8] described implementing an asynchronous communication platform for healthcare professionals to save time compared to synchronous communication. Moreover, Tran et al. explained that primary care providers requested more than a generic approach to a common clinical scenario when they contacted a specialist via an e-consultant application designed for interaction between healthcare professionals [10]. Written asynchronous communication between healthcare professionals and patients during treatment was experienced in favourable terms by the patients regarding freedom, security and certainty. This was closely linked to patients stressing the advantages that the healthcare professionals know who they are. The patients also point to the importance of being familiar with the healthcare professionals [9]. Entezarjou et al. [13] explored the staff's experience of an asynchronous chat for healthcare professionals and patients. Several benefits of the system were described, such as an increased opportunity for the patients to express themself as well as aid in triage by giving an overview of incoming presenting symptoms. For example, concerns about the system included the staff feeling involuntarily responsible for irrelevant symptoms reported by the platform and being overwhelmed with information for clinical decision making [13]. In addition, the implementation affected practice organisation and working environment. It can become problematic when IT systems are developed without considering the skills used by those involved in patient care [11]. Other studies have also focused on the work engagement aspects of asynchronous chat systems. In this study, nurses experienced less time- and emotional pressure and a decline in job control and feedback from co-workers working from home [12] when implementing such a system.

Even if technical innovations and automation are introduced in health care, the core of the patient-centred work for the care staff remains, including tacit knowledge, professional reflective practice and professional autonomy [14–16]. However, little is known about how increased automation in patient contact affects the healthcare staff’s professional autonomy and decision making. Hence this study aims to understand how occupational professionalism and the possibility of professional judgment are affected when clinical patient contact is digitised. A project where nurses and physicians used a chat program for communication with patients is the basis, and we analyse professional judgment and professionalism with the use of professionalism theory. The study’s overall question concerns whether and how the scope of the healthcare staff's professional judgment and occupational professionalism are affected by digitalisation.

2. Professionalism and discretionary decision making

How professions should be defined from other occupations has long been debated [17,18]. Burrage et al. presented a definition of professionalism in the 1990s [19], which was further developed in the early 2000s [20]: There is often a high degree of science in knowledge development for professionals, but professional practice can also be based on other knowledge, called proven experience. Moreover, professional practice is often characterised by independence; it is the professional who decides on adequate actions and has significant influence over his division of work. Traditionally, the professions have been challenging to govern, and the typical control has taken place by the members themselves collegially. The possibility of sanctions, for example, exclusions, exists as disciplinary measures.

Evetts distinguishes organisational professionalism and occupational professionalism [21]. Occupational professionalism represents the classic image of professional management, including collegial authority, self-government and collegial review [21]. However, occupational professionalism also includes space for manoeuvre for one's assessments and decision-making, so-called discretionary decision-making. This is in contrast to the so-called organisational professionalism. Instead, this professionalism focuses on legal-rational decision-making, where tasks tend to be standardised by regulatory systems and a low degree and opportunity for discretionary assessment/decision making. Evett's concepts have been summarised in Table 1 adapted from [22].

Table 1. Occupational Professionalism and Organisational Professionalism

**Occupational professionalism**	**Organisational professionalism**
control takes place within one's professional group	the striving for manager's power in the organisation
Collegial authority<br>Discretionary decision-making in complex cases<br>Professional control of the work<br>Trust from clients/patients and employees towards the practitioner / the professional.	Hierarchical structure regarding authority<br>Rational-legal decision-making<br>Standardised work process<br>Evaluation, goals, goal fulfilment

Evetts further points out that occupational professionalism and organisational professionalism are two ideal types and that the reality is not as pure as the models suggest. Evetts clarifies that organisational professionalism might threaten and undermine occupational professionalism [21,23]. There is a risk that professional values and norms are subject to market logic and commercial principles, resulting from increased bureaucratisation, reduced opportunities for professional ethics and decreasing opportunities for discretionary decision-making.

In Evett's descriptions, trust, discretionary decision-making, and competence are central to professional execution [21,23,24]. Other researchers agree that discretionary decision-making is major to professionals in their performance of tasks [25,26]. Although a high

degree of discretionary decision-making can be burdensome for professionals, this type of decision-making enables the exercise of professional judgment when organisations' rules are vague, contradictory or even harmful to clients/patients [27].

3. The Case Study

In 2003 the national service 1177 was established, initially providing online self-care information and healthcare advice via telephone from nurses 24 hours a day, all year round. Around 1200 nurses work full or part-time, assisting the 7 million people who call each year (from the 10 million inhabitants in Sweden). Approximately 30-35% of all calls are given self-care advice, whereas the rest are referred to a healthcare facility.

Recently, politicians in Uppsala decided to introduce a digital system to facilitate citizens' contact with healthcare providers. A proof of concept project was started in 2019, enabling asynchronous chat between citizens and nurses working at 1177 in Uppsala. The chat is operated by one to two nurses during the daytime and evenings. In addition, a physician is available during the last four staffed hours each day. The number of advice-seekers has increased during the project but not met the expected number.

The IT system in use obliges the patients to fill in a predefined questionnaire aimed at their specific contact cause from a list of available alternatives. The answers are presented to the nurses and physicians. When the patient has filled in the form, the healthcare personnel interact with the patient via the asynchronous written chat function.

The work of the nurses and physicians in the project differ in some aspects. The nurses are accustomed to giving medical advice in a human to human setting using a telephone. To aid in their work, they have an electronic decision support system with which they are familiar. Physicians' primary duty in the project is to provide medical advice, diagnose and treat the patients. If neither were deemed possible, the physicians would refer patients to a physical healthcare setting.

4. Method

Contextual inquiries [28] were conducted with five informants during one day to understand their work better. The researcher asked questions on how the work was done and took notes. This was followed by 17 semi-structured interviews (25-60 minutes) were conducted with nurses and physicians. A professional transcriber did transcriptions verbatim, and the authors analysed the transcribed material as described in Clark and Braun's thematic analysis method [29]. Patients and the public were not involved in the research work.

Nine interviews with nurses were conducted. The gender distribution was eight female and one male. The age range was 29-64 years, with an average age of 49 years. The average number of years as a professional nurse was 21.1 years (range 5-36 years). The number of years of working experience at 1177 was on average 3.4 years (range 1-11 years).

Moreover, four female physicians and four male physicians were interviewed in the age range of 26-49 years, with an average age of 34 years. Professional experience was two

For peer review only - http://bmjopen.bmj.com/site/about/guidelines.xhtml

specialised and six residents in general practice. No one had previous experience of having worked in 1177.

Patient and Public Involvement

This study did not include any patient involvement.

5. Result

Decision basis with questions to patients not tailored for their work

Both nurses and physicians express concerns about the predefined questionnaires for the patients to fill in before communicating with the healthcare personnel via the asynchronous chat. The system automates these questions. The healthcare personnel cannot see what questions the system asks the patients and how they are phrased. They experience that the decision basis with questions to patients is not tailored for their work as they can only see the answers from the patients. When the patients have started the asynchronous written chat, only later in the contact comes an opportunity to ask the questions they consider relevant to the health issues problems that the patients describe. However, several nurses experience the lack of knowledge about how questions are phrased created uncertainty about how they should interpret the answers and what further questions to ask.

"And then we can't see what the questions are in the questionnaire, so what exactly should I ask? What, what questions do I ask with this new questionnaire?" (Nurse 2).

The nurses and physicians express that the questionnaires are not perceived as tailored and adapted to healthcare counselling activities or triaging or determining patients' treatments' priority but are generally directed at health care. Consequently, many of the questions are perceived as irrelevant.

"It is not developed and directed for our needs, but may generally work for healthcare. Eh… so it is for example that eh ... many questionnaires that the patient can choose when they seek care are not relevant to us." (Nurse 2).

The physicians also express different views within the medical profession regarding what is considered possible in digital care. Sometimes, it seems as if others think they should take care of more than what is possible.

"... it is probably SKL, I think, Sweden's municipality and county council that has come up with a list of what you can handle via video and chat." (Physician 4).

"... the medical profession is quite divided about what can be diagnosed, such as a bacterial sore throat. Eh, I think it would be tough to judge based on a written chat. I would say almost impossible." (Physician 4).

Reduced trust with written communication

The nurses express difficulties in trusting what patients state in text and checkboxes. They relate this to a more complex evaluation and understanding of the information compared with telephone contact. Also, some nurses claim that the instruction is not to ask follow-up questions since this is time-consuming and should base their judgment on the information provided in the written chat. However, they feel that even though they need to ask more follow-up questions, uncertainty can remain.

"But I think it's because we're so insecure. We want more information before we make an assessment. We are not confident enough in the assessments we make. Because we want to ask more follow-up questions as we do on the phone." (Nurse 6).

One example highlighted by the nurses was that the forms provide information that they must consider, such as chest pain, even if that was not what the patient described as the cause of the contact. They describe uncertainty regarding this information since there is no information about when the symptoms occurred. It thus becomes difficult for nurses to assess the severity of the symptoms.

"... which you have to ask about; 'is it true that you have palpitations and how long have you had it?' And then there may be something like 'Yes, I've had it for two years, it's nothing new', but I do not see it. And must take it very seriously." (Nurse 9).

There is an expressed desire among the nurses to influence which questions the patient receives in the questionnaires significantly. Therefore, the nurses should have an opportunity to choose for themselves how and which questions to ask.

In comparison to the nurses, several physicians express a positive perspective regarding work and nurses triaging patients. The physicians feel they receive much information before contacting the patient, which is an effective way to work. However, they also experience it negatively to have very little influence over the questionnaires when there are shortcomings.

"It's also a thing like this when you have eh… the cause of cough for example. And nowhere is there the question of whether the patient is a smoker." (Physician 1).

Reduced opportunity to obtain information through chat communication

Several nurses describe aspects that are addressed that are lost in a chat contact compared to the telephone: Examples are the ability to hear any effect on the patient's breathing. They describe information such as hearing if the patient's breathing is heavy, hissing, strained or mushy speech, which could guide the assessment of ailments for counselling. Aspects such as the occurrence and differentiation of dry cough or mucus cough are also present in this. For example, one nurse describes that she wants to hear a person's breathing to assess whether the situation is urgent. The nurses also discuss the patients' estimates of pain and their information to interpret these estimates. For example, patients might state a very high level of pain on the phone, but being unaffected in the voice may not have severe pain as reported by the patient and not urgent with emergency interventions.

"You sound very unaffected in the voice and things like this and so on and then it becomes like this, then I can still hear 'no but it's probably not as serious as the patient states." (Nurse 1).

This type of information is described as disappearing when working with the chat in comparison with a telephone. As a result, there is only the patient's graded pain in the written conversation, and it becomes more difficult for the nurses to make emergency assessments.

Both nurses and physicians also compare the possibility of making an assessment based on information from the chat and information available at a physical patient visit. For example, nurses describe that a physical patient visit allows controlling vital parameters such as blood pressure, heart rate, respiration. However, this is not possible when they chat with the patients. Therefore, several nurses describe the physical visits, meet and see the patient, and do the previously mentioned examinations and checks to be the best alternative for assessing healthcare.

The physicians' stories describe limitations and difficulties in their assessments based on a chat contact. Physicians perceive it difficult when they do not receive answers to their questions. If they receive responses, they become uncertain what to do with answers that are not relevant based on the patient's search cause but potentially may be medically appropriate. In the chat, the assessment basis becomes what the patient chooses to tell. Physicians express that it is more difficult to determine whether all relevant information is provided or not in the chat. They compare these situations to physical visits where they can depend on both the conversation and the physical examination.

"So, it is also the patient's responsibility to a certain extent. I mean, I can only judge from what you say to me over the chat and I can only judge from the outside as physical and what you get in the conversation in the examination room. What happens in 24 hours, I do not know?" (Physician 7).

It appears that physicians see the chat as a compliment in many ways but not able to replace physical visits fully. Physicians express that it is only a small number of conditions that they can assess with certainty. Compared with physical visits, much of the information they need in their assessments and decision choices disappear in the chat.

"My opinion is that video and chat can't replace a physical visit with the same high quality. There are so many parameters that can make the quality and assessment better with a physical visit." (Physician 4).

Like the nurses, physicians describe aspects such as assessing how a person speaks and moves. Some parameters can be small but crucial to warn that something is not right and not perceived in a chat conversation. Also, the information in the text becomes more difficult to interpret when it comes to specific symptoms.

"But I think that like it is much harder to understand a stomach with just words like this 'stomach ache' than if they were sitting in front of you and pointing or like more explained

For peer review only - http://bmjopen.bmj.com/site/about/guidelines.xhtml

because it should be written a lot if you are to understand how the stomach ache because it can be so incredibly different while yes but fever, fever is a fever after all." (physician 6).

6. Discussion

Even though both nurses and physicians think the predefined forms provide much information before meetings, there are different opinions if they are time-saving or not. These different opinions could be due to different approaches in dealing with the current situation. Both nurses and physicians describe that they have little insight into how and which questions are asked to patients via the predefined forms. Hence, they have two alternatives; rely on the system, although they express that the information given is not of the same quality as the information provided in a conversation, or repeat the majority of questions since an interpretation of an answer without knowing the question is difficult, which is the case with the predefined forms. They also said that the forms are not aimed at their activities, and they felt that they had minimal opportunity to influence the content of the forms. The problem with little contact between the IT department and the health care professionals is well-known [30,31] and is a root cause of this problem.

An IT system forcing healthcare staff to perform the work within a more limited framework is an example of when occupational professionalism turns into organisational professionalism, as described by [22–24]. They express increased organisational professionalism as healthcare professionals lose control and influence over essential elements of their professional practice and are expected to follow a standardised structure in which they have little insight.

The nurses have difficulties trusting what patients state in text and checkboxes. They experience they can make a more holistic evaluation and understanding of the information in a telephone conversation. Also, some nurses claim that the instruction is not to ask follow-up questions since this is time-consuming, and they should base their judgment on the information provided in the written chat. However, they feel that even though they need to ask more follow-up questions, uncertainty can remain. The issue of trust and automated systems is well-known in other areas. Research has shown that the professionals' trust increases if more information is provided by the system [32,33].

One example highlighted by the nurses was that the forms provide information that they must consider, such as chest pain, even if that was not what the patient described as the cause of the contact. They describe uncertainty regarding this information since there is no information about when the symptoms occurred and their severity. It thus becomes difficult for nurses to assess the severity of the symptoms. This is also an example of these standardised forms and questions resulting in too much information. This is consistent with previous research [34] that too much information overturns rather than helps in assessments and decisions. More data reveals a higher degree of complexity that the professional also needs to take into account. Klein [34] states that it is not always more or better information required to make professional decisions and reduce uncertainty. On the contrary, more intake can increase complexity and thus lead to more significant uncertainty.

For peer review only - http://bmjopen.bmj.com/site/about/guidelines.xhtml

The nurses described difficulties in assessments of potentially urgent cases. It is difficult to interpret and understand, for example, patients' estimates of pain or the occurrence of breathing difficulties. The physicians report problems in evaluating symptoms and limited opportunities to make reliable decisions. Some information they would need for this is not available more than in some specific and straightforward cases. Both nurses and physicians express that chat contact cannot correspond to a physical visit. Physicians describe perceived expectations from others that chat contact should be compared to a visit to a health centre, whereas nurses mainly relate to differences from telephone counselling.

At the same time, as the possibility for nurses and physicians' discretionary decision making decreases, they remain legally responsible for their choices. Who is accountable for the risks that result from the loss of information, and how much should healthcare professionals endure the uncertainty that a digital patient contact generates.

The scope for professional autonomy and discretionary decision-making is reduced through the introduction of the chat system. Still, the advantage is that by adhering to predetermined procedures, professionals can protect themselves against possible criticism, and in this experience, feel more confident in their decisions. Standardisations can be seen to stand for a professional, scientific execution, free from morality and subjective interpretations, and in this, be more clearly customer- or user-oriented [35]. A disadvantage of this, in turn, is that professionals may be less likely to take risks in their professional practice: that it becomes more important to follow the standardisations that exist, with the consequence that professional values and professional independence are left behind.

Ethics statement

The national ethical vetting authority ethically approved the study with the number 2019-04991.

Contributorship statement

ÅC and ML designed the study and did the data collection. ÅC, SL and GH analyzed the data. All authors contributed to the background sections, wrote the paper, and reviewed the manuscript.

Competing Interests

There are no competing interests

Funding

Open access funding provided by Uppsala University. AFA funded the research study through the project System Development Methods for a Digital Work Environment (STRIA).

Data sharing

No additional data available

References

1. Abdullah R, Fakieh B. Health Care Employees' Perceptions of the Use of Artificial

Intelligence Applications: Survey Study. Journal of medical Internet research. 2020 May 14;22(5):e17620.
2. Davenport T, Kalakota R. The potential for artificial intelligence in healthcare. Future Healthc J. 2019 Jun;6(2):94–8.
3. Eden R, Burton-Jones A, Scott I, Staib A, Sullivan C. Effects of eHealth on hospital practice: synthesis of the current literature. Aust Health Review. 2018;42(5):568.
4. Keasberry J, Scott IA, Sullivan C, Staib A, Ashby R. Going digital: a narrative overview of the clinical and organisational impacts of eHealth technologies in hospital practice. Aust Health Review. 2017;41(6):646.
5. Senbekov M, Saliev T, Bukeyeva Z, Almabayeva A, Zhanaliyeva M, Aitenova N, et al. The Recent Progress and Applications of Digital Technologies in Healthcare: A Review. Fayn J, editor. International Journal of Telemedicine and Applications. 2020 Dec 3;2020:1–18.
6. Wass S, Vimarlunda V. I got 99 problems, and eHealth is one. In: MEDINFO 2017: Precision Healthcare Through Informatics: Proceedings of the 16th World Congress on Medical and Health Informatics. IOS Press; 2018. p. 258.
7. Carayon P, Hoonakker P. Human factors and usability for health information technology: old and new challenges. Yearbook of medical informatics. 2019;28(01):071–7.
8. Jhala M, Menon R. Examining the impact of an asynchronous communication platform versus existing communication methods: an observational study. BMJ innovations. 2021;7(1).
9. Bjoernes CD, Laursen BS, Delmar C, Cummings E, Nøhr C. A dialogue-based web application enhances personalized access to healthcare professionals–an intervention study. BMC medical informatics and decision making. 2012;12(1):1–14.
10. Tran C, Archibald D, Humphrey-Murto S, Liddy C, Keely E. What makes a high-quality electronic consultation (eConsult)? A nominal group study. Journal of telemedicine and telecare. 2020;26(4):239–47.
11. Lintern G, Motavalli A. Healthcare information systems: the cognitive challenge. BMC Med Inform Decis Mak. 2018 Dec;18(1):3.
12. Cajander Å, Larusdottir M, Hedström G. The effects of automation of a patient-centric service in primary care on the work engagement and exhaustion of nurses. Quality and User Experience. 2020;5(1):1–13.
13. Entezarjou A, Bolmsjö BB, Calling S, Midlöv P, Nymberg VM. Experiences of digital communication with automated patient interviews and asynchronous chat in Swedish primary care: a qualitative study. BMJ open. 2020;10(7):e036585.
14. Schön DA. The reflective practitioner: How professionals think in action. Routledge; 2017.
15. Argyris C. Tacit knowledge and management. Tacit knowledge in professional practice. 1999;123–40.
16. Argyris C, Schon DA. Theory in practice: Increasing professional effectiveness. Jossey-bass; 1974.
17. Brante T. Professions as science-based occupations. Professions and professionalism. 2011;1(1).
18. Saks M. Defining a profession: The role of knowledge and expertise. Professions and Professionalism. 2012;2(1).
19. Burrage M, Jarausch K, Siegrist H. An actor-based framework for the study of the professions. Professions in theory and history: Rethinking the study of the professions. 1990;203–25.
20. Siegrist H. Professionalization/professions in history. 2001;
21. Evetts J. Professions in turbulent times: Changes, challenges and opportunities. Sociologia, Problemas e Práticas. 2018;(88):43–59.
22. Öberg SA, Bringselius L. Professionalism and organizational performance in the wake of new managerialism. European Political Science Review: EPSR. 2015;7(4):499.
23. Evetts J. New professionalism and new public management: Changes, continuities and

For peer review only - http://bmjopen.bmj.com/site/about/guidelines.xhtml

consequences. Comparative sociology. 2009;8(2):247–66.
24. Evetts J. Introduction: Trust and professionalism: Challenges and occupational changes. SAGE London, Thousand Oaks, CA and New Delhi; 2006.
25. Lipsky M. Street-level bureaucracy: Dilemmas of the individual in public service. Russell Sage Foundation; 2010.
26. Wallander L, Molander A. Disentangling professional discretion: A conceptual and methodological approach. 2014;
27. Ponnert L, Svensson K. Standardisation—the end of professional discretion? European Journal of Social Work. 2016;19(3–4):586–99.
28. Holtzblatt K, Beyer H. Contextual design: Design for life. Morgan Kaufmann; 2016.
29. Braun V, Clarke V. Successful qualitative research: A practical guide for beginners. sage; 2013.
30. Golay D, Cajander \AAsa, Hussein D, Azeez A, Bonacina S. Communication Breakdowns between Nurses and IT Department: Why Hospitals Fail at Improving the Usability of Health Information Technology. In: International Symposium on Health Information Management Research. 2020.
31. Shah SGS, Robinson I. User involvement in healthcare technology development and assessment. International Journal of Health Care Quality Assurance. 2006;
32. Verbeke P, Verguts T. Learning to synchronize: How biological agents can couple neural task modules for dealing with the stability-plasticity dilemma. PLoS computational biology. 2019 Aug 20;15(8):e1006604.
33. Verberne FMF, Ham J, Midden CJH. Trust in smart systems: sharing driving goals and giving information to increase trustworthiness and acceptability of smart systems in cars. Human factors. 2012 Oct;54(5):799–810.
34. Klein GA. Streetlights and shadows: Searching for the keys to adaptive decision making. MIT Press; 2011.
35. Barfoed EM, Jacobsson K. Moving from ‘gut feeling’to ‘pure facts’: Launching the ASI interview as part of in-service training for social workers. Nordic Social Work Research. 2012;2(1):5–20.

For peer review only - http://bmjopen.bmj.com/site/about/guidelines.xhtml

BMJ Open

Professional Decision Making with Digitalisation of Patient Contacts in a Medical Advice Setting: A Qualitative Study of a Pilot Project with a Chat Program in Sweden

Journal:	*BMJ Open*
Manuscript ID	bmjopen-2021-054103.R1
Article Type:	Original research
Date Submitted by the Author:	26-Oct-2021
Complete List of Authors:	Cajander, Åsa; Uppsala Universitet Teknisk-naturvetenskapliga fakulteten, Department of Information Technology<br>Hedström , Gustaf ; Uppsala Universitet, Department of Public Health and Caring Sciences<br>Leijon , Sofia ; Uppsala Universitet, Department of Information Technology<br>Larusdottir , Marta; Reykjavik University School of Computer Science, School of Computer Science
**Primary Subject Heading**:	Health informatics
Secondary Subject Heading:	Nursing, Public health, Qualitative research
Keywords:	Health informatics < BIOTECHNOLOGY & BIOINFORMATICS, Information technology < BIOTECHNOLOGY & BIOINFORMATICS, Telemedicine < BIOTECHNOLOGY & BIOINFORMATICS, Human resource management < HEALTH SERVICES ADMINISTRATION & MANAGEMENT, Organisation of health services < HEALTH SERVICES ADMINISTRATION & MANAGEMENT, Public health < INFECTIOUS DISEASES

SCHOLARONE™
Manuscripts

I, the Submitting Author has the right to grant and does grant on behalf of all authors of the Work (as defined in the below author licence), an exclusive licence and/or a non-exclusive licence for contributions from authors who are: i) UK Crown employees; ii) where BMJ has agreed a CC-BY licence shall apply, and/or iii) in accordance with the terms applicable for US Federal Government officers or employees acting as part of their official duties; on a worldwide, perpetual, irrevocable, royalty-free basis to BMJ Publishing Group Ltd ("BMJ") its licensees and where the relevant Journal is co-owned by BMJ to the co-owners of the Journal, to publish the Work in this journal and any other BMJ products and to exploit all rights, as set out in our licence.

The Submitting Author accepts and understands that any supply made under these terms is made by BMJ to the Submitting Author unless you are acting as an employee on behalf of your employer or a postgraduate student of an affiliated institution which is paying any applicable article publishing charge ("APC") for Open Access articles. Where the Submitting Author wishes to make the Work available on an Open Access basis (and intends to pay the relevant APC), the terms of reuse of such Open Access shall be governed by a Creative Commons licence – details of these licences and which Creative Commons licence will apply to this Work are set out in our licence referred to above.

Other than as permitted in any relevant BMJ Author's Self Archiving Policies, I confirm this Work has not been accepted for publication elsewhere, is not being considered for publication elsewhere and does not duplicate material already published. I confirm all authors consent to publication of this Work and authorise the granting of this licence.

For peer review only

Professional Decision Making with Digitalisation of Patient Contacts in a Medical Advice Setting: A Qualitative Study of a Pilot Project with a Chat Program in Sweden

Åsa Cajander[1], Gustaf Hedström[2], Sofia Leijon[1], Marta Larusdottir[3]

1.Department of Information Technology, Uppsala University
2.Department of Public Health and Caring Sciences, Uppsala University
3.Department of Computer Science, Reykjavik University

Corresponding author:
Åsa Cajander, Uppsala University, Lägerhyddsvägen 2, Box 337, 751 05 Uppsala
Email: asa.cajander@it.uu.se
Phone: 0704-250 786

Key Words: automation, eHealth patient-centric services, decision-making, assessment, healthcare professionals

Word count: 3657

Abstract

Objectives: Patient e-services are increasingly launched globally to make health care more efficient and digitalised. One area that is digitalised is medical advice, where patients asynchronously chat with nurses and physicians, with patients having filled in a form with predefined questions before the chat. This study aimed to explore how occupational professionalism and the possibility of professional judgment are affected when clinical patient contact is digitalised. The study's overall question concerns whether and how the scope of the healthcare staff's professional judgment and occupational professionalism are affected by digitalisation.
Design and setting: A qualitative study of health care professionals working in a pilot project with a chat program for patients in a medical advice setting in Sweden.
Participants and analysis: Contextual inquiries and 17 interviews with nurses (n=9) and physicians (n=8). The interviews were thematically analysed. The analysis was inductive and based on theories of decision making.
Results: Three themes emerged: (1) Predefined questions to patients not tailored for health care professionals' work (2) reduced trust in written communication, and (3) reduced opportunity to obtain information through chat communication.
Conclusions: The results indicate that asynchronous chat with patients might reduce the opportunity for nurses and physicians to obtain and use professional knowledge and discretionary decision-making. Furthermore, the system's design increases uncertainty in assessments and decision-making, which reduces the range of occupational professionalism.

Strengths and limitations of this study

- A methodological strength of the study is the combination of field studies with an interview study
- Another methodological strength is that this case study is based on an implementation project and not a research intervention.
- A further strength of the study is the unique and highly relevant research question that has not been explored before.
- A limitation of the study is the limited numbers of nurses and physicians in the interview study.

1. Introduction

The idea that new technology will solve current or future difficulties at work is not new. The digitalisation of healthcare is no exception when new technologies such as AI have been highlighted as solutions to existing problems [1,2]. However, follow-ups have shown that the systems rarely deliver the expected benefits regarding, among other things, increased efficiency and safety [3–6]. Moreover, the results from eHealth implementations vary depending on the solution and organisational context [7].

One recent area with varying implementation results is asynchronous communication platforms where health care professionals and patients interact [8–12]. For example, Jhala and Menon [8] described implementing an asynchronous communication platform for healthcare professionals to save time compared to synchronous communication. Moreover, Tran et al. explained that primary care providers requested more than a generic approach to a common clinical scenario when they contacted a specialist via an e-consultant application designed for interaction between healthcare professionals [10]. Written asynchronous communication between healthcare professionals and patients during treatment was experienced in favourable terms by the patients regarding freedom, security and certainty. This was closely linked to patients stressing the advantages that the healthcare professionals know who they are. The patients also point to the importance of being familiar with the healthcare professionals [9]. Entezarjou et al. [13] explored the staff's experience of an asynchronous chat for healthcare professionals and patients. Several benefits of the system were described, such as an increased opportunity for the patients to express themself as well as aid in triage by giving an overview of incoming presenting symptoms. For example, concerns about the system included the staff feeling involuntarily responsible for irrelevant symptoms reported by the platform and being overwhelmed with information for clinical decision making [13]. In addition, the implementation affected practice organisation and working environment. It can become problematic when IT systems are developed without considering the skills used by those involved in patient care [11]. Other studies have also focused on the work engagement aspects of asynchronous chat systems. In one study, nurses experienced less time- and emotional pressure and a decline in job control and feedback from co-workers working from home [12] when implementing such a system.

How professions should be differentiated from other occupations has long been debated [14,15]. Burrage et al. presented a definition of professionalism in the 1990s [16], which was further developed in the early 2000s [17]: There is often a high degree of science in knowledge development for professionals, but professional practice can also be based on other knowledge, called proven experience. Moreover, professional practice is often characterised by independence; it is the professional who decides on adequate actions and has significant influence over his division of work. Traditionally, the professions have been challenging to govern, and the typical control has taken place by the members themselves collegially. The possibility of sanctions, for example, exclusions, exists as disciplinary measures.

Evetts distinguishes organisational professionalism and occupational professionalism [18]. Occupational professionalism represents the classic image of professional management, including collegial authority, self-government and collegial review [18]. However, occupational professionalism also includes space for manoeuvre for one's assessments and decision-making, so-called discretionary decision-making. Moreover, control takes place within one's professional group and occupational professionalism is based on collegial authority with professionals in control of the work. This is in contrast to the so-called organisational professionalism. Instead, this professionalism focuses on legal-rational decision-making, where tasks tend to be standardised by regulatory systems and a low degree and opportunity for discretionary assessment/decision making. In the organisational

For peer review only - http://bmjopen.bmj.com/site/about/guidelines.xhtml

professionalism paradigm, there are hierarchical structures regarding authority and a striving for managers' power in the organisation. In this way of organising work evaluations, goals and goals fulfilment are common. Evetts further points out that occupational professionalism and organisational professionalism are two ideal types and that the reality is not as pure as the models suggest. Evetts clarifies that organisational professionalism might threaten and undermine occupational professionalism [18,19]. There is a risk that professional values and norms are subject to market logic and commercial principles, resulting from increased bureaucratisation, reduced opportunities for professional ethics and decreasing opportunities for discretionary decision-making. In Evett's descriptions, trust, discretionary decision-making, and competence are central to professional execution [18–20]. Other researchers agree that discretionary decision-making is major to professionals in their performance of tasks [21,22]. Although a high degree of discretionary decision-making can be burdensome for professionals, this type of decision-making enables the exercise of professional judgment when organisations' rules are vague, contradictory or even harmful to clients/patients [23].

Even if technical innovations and automation are introduced in health care, the core of the patient-centred work for the care staff remains, including tacit knowledge, professional reflective practice and professional autonomy [24–26]. However, little is known about how increased automation in patient contact affects the healthcare staff's professional autonomy and decision making.

Hence this study aims to understand how occupational professionalism and the possibility of professional judgment are affected when clinical patient contact is digitalised. A project where nurses and physicians used a chat program for communication with patients is the basis, and we analyse professional judgment and professionalism with the use of professionalism theory. The study's overall question concerns whether and how the scope of the healthcare staff's professional judgment and occupational professionalism are affected by this digitalisation.

2. Methods

2.1 Contextual setting

In 2003 the national service 1177 was established, initially providing online self-care information and healthcare advice via telephone from nurses 24 hours a day, all year round. Around 1200 nurses work full or part-time, assisting the 7 million people who call each year (from the 10 million inhabitants in Sweden). Approximately 30-35% of all calls are given self-care advice, whereas the rest are referred to a healthcare facility.

Recently, politicians in Uppsala decided to introduce a digital system to facilitate citizens' contact with healthcare providers. A proof-of-concept project was started in 2019, enabling asynchronous chat between citizens and nurses working at 1177 in Uppsala. The chat is operated by one to two nurses during the daytime and evenings. In addition, a physician is available during the last four staffed hours each day. The number of advice-seekers has increased during the project but not met the expected number.

The IT system in use obliges the patients to fill in a predefined questionnaire aimed at their specific chief complaint from a list of available alternatives. The answers are presented to the nurses and physicians. When the patient has filled in the form, the healthcare personnel interact with the patient via the asynchronous written chat function.

The work of the nurses and physicians in the project differ in some aspects. The nurses are accustomed to giving medical advice in a human-to-human setting using a telephone. To aid in their work, they have an electronic decision support system with which they are familiar. Physicians' primary duty in the project is to provide medical advice, diagnose and treat the patients. If neither were deemed possible, the physicians would refer patients to a physical healthcare setting.

For peer review only - http://bmjopen.bmj.com/site/about/guidelines.xhtml

2.2 Qualitative methods
In this qualitative study contextual inquiries [27] were conducted with five informants during one day to understand their work better. The researcher asked questions on how the work was done and took notes. The contextual inquiries were used to understand the context of the work and as background information.

This was followed by 17 semi-structured interviews (25-60 minutes) conducted with nurses (n=9) and physicians (n=8; see supplementary file for the interview template). All participants from the contextual inquiries and the interviews were asked to provide written informed consent.

A professional transcriber did transcriptions verbatim, and the authors analysed the transcribed interviews as described in Clark and Braun's thematic analysis method [28]. We used a theoretical framework of occupational professionalism as presented in the introduction for the interpretation of the results.

Patient and Public Involvement

This study did not include any patient or public involvement.

3. Results

Nine interviews with nurses were conducted. The gender distribution was eight females and one male. The age range was 29-64 years, with an average age of 49 years. The average number of years as a professional nurse was 21.1 years (range 5-36 years). The number of years of working experience at 1177 was on average 3.4 years (range 1-11 years).

Moreover, four female physicians and four male physicians were interviewed in the age range of 26-49 years, with an average age of 34 years. Professional experience was two specialists and six residents in general practice. No one had previous experience of having worked in 1177.

Predefined questions to patients not tailored for health care professionals' work

Both nurses and physicians express concerns about the predefined questionnaires for the patients to fill in before communicating with the healthcare personnel via the asynchronous chat. The interviewees said that these questions were not the same questions as in the elderly telephone system, but were provided by the IT company and adapted to the 1177 context in workshops and were updated during the pilot project. The system automates these questions. The healthcare personnel cannot see what questions the system asks the patients and how they are phrased. They experience that the predefined questions to patients is not tailored for their work as they can only see the answers from the patients. When the patients have started the asynchronous written chat, only later in the contact comes an opportunity to ask the questions they consider relevant to the health issues problems that the patients describe. However, several nurses experience the lack of knowledge about how questions are phrased created uncertainty about how they should interpret the answers and what further questions to ask.

"And then we can’t see what the questions are in the questionnaire, so what exactly should I ask? What, what questions do I ask with this new questionnaire?” (Nurse 2).

For peer review only - http://bmjopen.bmj.com/site/about/guidelines.xhtml

The nurses and physicians express that the questionnaires are not perceived as tailored and adapted to healthcare counselling activities or triaging or determining patients' treatments' priority but are generally directed at health care. Consequently, many of the questions are perceived as irrelevant.

“It is not developed and directed for our needs, but may generally work for healthcare. Eh… so it is for example that eh ... many questionnaires that the patient can choose when they seek care are not relevant to us.” (Nurse 2).

The physicians also express different views within the medical profession regarding what is considered possible in digital care. Sometimes, it seems as if others think they should take care of more than what is possible.

"... it is probably Sweden's municipality and county council that has come up with a list of what you can handle via video and chat." (Physician 4).

“... the medical profession is quite divided about what can be diagnosed, such as a bacterial sore throat. Eh, I think it would be tough to judge based on a written chat. I would say almost impossible.” (Physician 4).

Reduced trust with written communication

The nurses express difficulties in trusting what patients state in text and checkboxes. They relate this to a more complex evaluation and understanding of the information compared with telephone contact. Also, some nurses claim that the instruction is not to ask follow-up questions since this is time-consuming and should base their judgment on the information provided in the written chat. However, they feel that even though they need to ask more follow-up questions, uncertainty can remain.

"But I think it's because we're so insecure. We want more information before we make an assessment. We are not confident enough in the assessments we make. Because we want to ask more follow-up questions as we do on the phone.” (Nurse 6).

One example highlighted by the nurses was that the forms provide information that they must consider, such as chest pain, even if that was not what the patient described as the cause of the contact. They describe uncertainty regarding this information since there is no information about when the symptoms occurred. It thus becomes difficult for nurses to assess the severity of the symptoms.

“... which you have to ask about; 'is it true that you have palpitations and how long have you had it?' And then there may be something like 'Yes, I've had it for two years, it's nothing new', but I do not see it. And must take it very seriously." (Nurse 9).

There is an expressed desire among the nurses to influence which questions the patient receives in the questionnaires significantly. Therefore, the nurses should have an opportunity to choose for themselves how and which questions to ask.

In comparison to the nurses, several physicians express a positive perspective regarding work and nurses triaging patients. The physicians feel they receive much information before contacting the patient, which is an effective way to work. However, they also experience it negatively to have very little influence over the questionnaires when there are shortcomings.

“It's also a thing like this when you have eh… the cause of cough for example. And nowhere is there the question of whether the patient is a smoker.” (Physician 1).

For peer review only - http://bmjopen.bmj.com/site/about/guidelines.xhtml

Reduced opportunity to obtain information through chat communication

Several nurses describe aspects that are addressed that are lost in a chat contact compared to the telephone: Examples are the ability to hear any effect on the patient's breathing. They describe information such as hearing if the patient's breathing is heavy, hissing, strained or mushy speech, which could guide the assessment of ailments for counselling. Aspects such as the occurrence and differentiation of dry cough or mucus cough are also present in this. For example, one nurse describes that she wants to hear a person’s breathing to assess whether the situation is urgent. The nurses also discuss the patients' estimates of pain and their information to interpret these estimates. For example, patients might state a very high level of pain on the phone, but being unaffected in the voice may not have severe pain as reported by the patient and not urgent with emergency interventions.

"You sound very unaffected in the voice and things like this and so on and then it becomes like this, then I can still hear 'no but it's probably not as serious as the patient states.” (Nurse 1).

This type of information is described as disappearing when working with the chat in comparison with a telephone. As a result, there is only the patient's graded pain in the written conversation, and it becomes more difficult for the nurses to make emergency assessments.

Both nurses and physicians also compare the possibility of making an assessment based on information from the chat and information available at a physical patient visit. For example, nurses describe that a physical patient visit allows controlling vital parameters such as blood pressure, heart rate, respiration. However, this is not possible when they chat with the patients. Therefore, several nurses describe the physical visits, meet and see the patient, and do the previously mentioned examinations and checks to be the best alternative for assessing healthcare.

The physicians' stories describe limitations and difficulties in their assessments based on a chat contact. Physicians perceive it difficult when they do not receive answers to their questions. If they receive responses, they become uncertain what to do with answers that are not relevant based on the patient's search cause but potentially may be medically appropriate. In the chat, the assessment basis becomes what the patient chooses to tell. Physicians express that it is more difficult to determine whether all relevant information is provided or not in the chat. They compare these situations to physical visits where they can depend on both the conversation and the physical examination.

“So, it is also the patient's responsibility to a certain extent. I mean, I can only judge from what you say to me over the chat and I can only judge from the outside as physical and what you get in the conversation in the examination room. What happens in 24 hours, I do not know?” (Physician 7).

It appears that physicians see the chat as a compliment in many ways but not able to replace physical visits fully. Physicians express that it is only a small number of conditions that they can assess with certainty. Compared with physical visits, much of the information they need in their assessments and decision choices disappear in the chat.

“My opinion is that video and chat can’t replace a physical visit with the same high quality. There are so many parameters that can make the quality and assessment better with a physical visit.” (Physician 4).

Like the nurses, physicians describe aspects such as assessing how a person speaks and moves. Some parameters can be small but crucial to warn that something is not right and

not perceived in a chat conversation. Also, the information in the text becomes more difficult to interpret when it comes to specific symptoms.

"But I think that like it is much harder to understand a stomach with just words like this 'stomach ache' than if they were sitting in front of you and pointing or like more explained because it should be written a lot if you are to understand how the stomach ache because it can be so incredibly different while yes but fever, fever is a fever after all." (physician 6).

4. Discussion

Even though both nurses and physicians think the predefined forms provide much information before meetings, there are different opinions if they are time-saving or not. These different opinions could be due to different approaches in dealing with the current situation. Both nurses and physicians describe that they have little insight into how and which questions are asked to patients via the predefined forms. Hence, they have two alternatives; rely on the system, although they express that the information given is not of the same quality as the information provided in a conversation, or repeat the majority of questions since an interpretation of an answer without knowing the question is difficult, which is the case with the predefined forms. They also said that the forms are not aimed at their activities, and they felt that they had minimal opportunity to influence the content of the forms. The problem with little contact between the IT department and the health care professionals is well-known [29,30] and is a root cause of this problem.

An IT system forcing healthcare staff to perform the work within a more limited framework is an example of when occupational professionalism turns into organisational professionalism, as described by [19,20,31]. They express increased organisational professionalism as healthcare professionals lose control and influence over essential elements of their professional practice and are expected to follow a standardised structure in which they have little insight.

The nurses have difficulties trusting what patients state in text and checkboxes. They experience they can make a more holistic evaluation and understanding of the information in a telephone conversation. Also, some nurses claim that the instruction is not to ask follow-up questions since this is time-consuming, and they should base their judgment on the information provided in the written chat. However, they feel that even though they need to ask more follow-up questions, uncertainty can remain. The issue of trust and automated systems is well-known in other areas. Research has shown that the professionals' trust increases if more information is provided by the system [32,33].

One example highlighted by the nurses was that the forms provide information that they must consider, such as chest pain, even if that was not what the patient described as the cause of the contact. They describe uncertainty regarding this information since there is no information about when the symptoms occurred and their severity. It thus becomes difficult for nurses to assess the severity of the symptoms. This is also an example of these standardised forms and questions resulting in too much information. This is consistent with previous research [34] that too much information overturns rather than helps in assessments and decisions. More data reveals a higher degree of complexity that the professional also needs to take into account. Klein [34] states that it is not always more or better information required to make professional decisions and reduce uncertainty. On the contrary, more intake can increase complexity and thus lead to more significant uncertainty.

The nurses described difficulties in assessments of potentially urgent cases. It is difficult to interpret and understand, for example, patients' estimates of pain or the occurrence of breathing difficulties. The physicians report problems in evaluating symptoms and limited

For peer review only - http://bmjopen.bmj.com/site/about/guidelines.xhtml

opportunities to make reliable decisions. Some information they would need for this is not available more than in some specific and straightforward cases. Both nurses and physicians express that chat contact cannot correspond to a physical visit. Physicians describe perceived expectations from others that chat contact should be compared to a visit to a health centre, whereas nurses mainly relate to differences from telephone counselling.

At the same time, as the possibility for nurses and physicians' discretionary decision making decreases, they remain legally responsible for their choices. Who is accountable for the risks that result from the loss of information, and how much should healthcare professionals endure the uncertainty that a digital patient contact generates.

The scope for professional autonomy and discretionary decision-making is reduced through the introduction of the chat system. Still, the advantage is that by adhering to predetermined procedures, professionals can protect themselves against possible criticism, and in this experience, feel more confident in their decisions. Standardisations can be seen to stand for a professional, scientific execution, free from morality and subjective interpretations, and in this, be more clearly customer- or user-oriented [35]. A disadvantage of this, in turn, is that professionals may be less likely to take risks in their professional practice: that it becomes more important to follow the standardisations that exist, with the consequence that professional values and professional independence are left behind.

Contributors

ÅC and ML did the conception and design of the study. ÅC and ML did the data collection. GH and SL did the analysis and interpretation of the data. SL drafted the first version of the manuscript. ÅC, ML, GH, and SL contributed to the interpretation, work with the finalisation of the manuscript and approved the final manuscript and agreed to be accountable for all aspects of the work in ensuring integrity and accuracy. The guarantor is ÅC.

Competing interests

There are no competing interests for any author

Ethics statement

The Swedish Ethical Review Authority ethically approved the study with the number 2019-04991.

Funding

This work was supported by AFA grant number 180250.

Data availability statement

No additional data available.

References

1. Abdullah R, Fakieh B. Health Care Employees' Perceptions of the Use of Artificial Intelligence Applications: Survey Study. Journal of medical Internet research. 2020 May 14;22(5):e17620.
2. Davenport T, Kalakota R. The potential for artificial intelligence in healthcare. Future Healthc J. 2019 Jun;6(2):94–8.

For peer review only - http://bmjopen.bmj.com/site/about/guidelines.xhtml

3. Eden R, Burton-Jones A, Scott I, Staib A, Sullivan C. Effects of eHealth on hospital practice: synthesis of the current literature. Aust Health Review. 2018;42(5):568.
4. Keasberry J, Scott IA, Sullivan C, Staib A, Ashby R. Going digital: a narrative overview of the clinical and organisational impacts of eHealth technologies in hospital practice. Aust Health Review. 2017;41(6):646.
5. Senbekov M, Saliev T, Bukeyeva Z, Almabayeva A, Zhanaliyeva M, Aitenova N, et al. The Recent Progress and Applications of Digital Technologies in Healthcare: A Review. Fayn J, editor. International Journal of Telemedicine and Applications. 2020 Dec 3;2020:1–18.
6. Wass S, Vimarlunda V. I got 99 problems, and eHealth is one. In: MEDINFO 2017: Precision Healthcare Through Informatics: Proceedings of the 16th World Congress on Medical and Health Informatics. IOS Press; 2018. p. 258.
7. Carayon P, Hoonakker P. Human factors and usability for health information technology: old and new challenges. Yearbook of medical informatics. 2019;28(01):071–7.
8. Jhala M, Menon R. Examining the impact of an asynchronous communication platform versus existing communication methods: an observational study. BMJ innovations. 2021;7(1).
9. Bjoernes CD, Laursen BS, Delmar C, Cummings E, Nøhr C. A dialogue-based web application enhances personalized access to healthcare professionals–an intervention study. BMC medical informatics and decision making. 2012;12(1):1–14.
10. Tran C, Archibald D, Humphrey-Murto S, Liddy C, Keely E. What makes a high-quality electronic consultation (eConsult)? A nominal group study. Journal of telemedicine and telecare. 2020;26(4):239–47.
11. Lintern G, Motavalli A. Healthcare information systems: the cognitive challenge. BMC Med Inform Decis Mak. 2018 Dec;18(1):3.
12. Cajander Å, Larusdottir M, Hedström G. The effects of automation of a patient-centric service in primary care on the work engagement and exhaustion of nurses. Quality and User Experience. 2020;5(1):1–13.
13. Entezarjou A, Bolmsjö BB, Calling S, Midlöv P, Nymberg VM. Experiences of digital communication with automated patient interviews and asynchronous chat in Swedish primary care: a qualitative study. BMJ open. 2020;10(7):e036585.
14. Brante T. Professions as science-based occupations. Professions and professionalism. 2011;1(1).
15. Saks M. Defining a profession: The role of knowledge and expertise. Professions and Professionalism. 2012;2(1).
16. Burrage M, Jarausch K, Siegrist H. An actor-based framework for the study of the professions. Professions in theory and history: Rethinking the study of the professions. 1990;203–25.
17. Siegrist H. Professionalization/professions in history. 2001;
18. Evetts J. Professions in turbulent times: Changes, challenges and opportunities. Sociologia, Problemas e Práticas. 2018;(88):43–59.
19. Evetts J. New professionalism and new public management: Changes, continuities and consequences. Comparative sociology. 2009;8(2):247–66.
20. Evetts J. Introduction: Trust and professionalism: Challenges and occupational changes. SAGE London, Thousand Oaks, CA and New Delhi; 2006.
21. Lipsky M. Street-level bureaucracy: Dilemmas of the individual in public service. Russell Sage Foundation; 2010.
22. Wallander L, Molander A. Disentangling professional discretion: A conceptual and methodological approach. 2014;
23. Ponnert L, Svensson K. Standardisation—the end of professional discretion? European Journal of Social Work. 2016;19(3–4):586–99.
24. Schön DA. The reflective practitioner: How professionals think in action. Routledge; 2017.
25. Argyris C. Tacit knowledge and management. Tacit knowledge in professional practice. 1999;123–40.

For peer review only - http://bmjopen.bmj.com/site/about/guidelines.xhtml

26. Argyris C, Schon DA. Theory in practice: Increasing professional effectiveness. Jossey-bass; 1974.
27. Holtzblatt K, Beyer H. Contextual design: Design for life. Morgan Kaufmann; 2016.
28. Braun V, Clarke V. Successful qualitative research: A practical guide for beginners. sage; 2013.
29. Golay D, Cajander \AAsa, Hussein D, Azeez A, Bonacina S. Communication Breakdowns between Nurses and IT Department: Why Hospitals Fail at Improving the Usability of Health Information Technology. In: International Symposium on Health Information Management Research. 2020.
30. Shah SGS, Robinson I. User involvement in healthcare technology development and assessment. International Journal of Health Care Quality Assurance. 2006;
31. Öberg SA, Bringselius L. Professionalism and organizational performance in the wake of new managerialism. European Political Science Review: EPSR. 2015;7(4):499.
32. Verbeke P, Verguts T. Learning to synchronize: How biological agents can couple neural task modules for dealing with the stability-plasticity dilemma. PLoS computational biology. 2019 Aug 20;15(8):e1006604.
33. Verberne FMF, Ham J, Midden CJH. Trust in smart systems: sharing driving goals and giving information to increase trustworthiness and acceptability of smart systems in cars. Human factors. 2012 Oct;54(5):799–810.
34. Klein GA. Streetlights and shadows: Searching for the keys to adaptive decision making. MIT Press; 2011.
35. Barfoed EM, Jacobsson K. Moving from ‘gut feeling’to ‘pure facts’: Launching the ASI interview as part of in-service training for social workers. Nordic Social Work Research. 2012;2(1):5–20.

For peer review only - http://bmjopen.bmj.com/site/about/guidelines.xhtml

Semi Structured Interview template

Information about the study

Who am I/are we that is/are conducting the interview?

The researchers carrying out the study are interested in digitalisation and work environment. The interview will cover different aspects of these topics.

There are no correct or incorrect answers. We want to learn from your experience. The interview will take around 45 minutes to 1 hour

Have you managed to read the information we sent you? If not we read the information.

Is there anything you would like to ask about?

Part 1: Background

Can you tell me a little about who you are? Allow the informant to talk first by him/herself, and then follow up with questions so that the following are covered:

(Gender), age
Can you describe your work situation now?
What is your job/profession?
How long have you been working at 1177?
Where did you work before?

For peer review only

Part 2: The chat

Now we will focus on how you work with the chat. I will ask you what it's like to use the system at work, so when you answer, I would like you to focus on your use of the systems and not on the problems the patients have.

Can you describe what you do when a patient you shall take care of enters the chat?
What systems do you use for chat during your working day?
Are there any differences between them for the user?
What systems do you use for chat most of the time?
What's it like to use Doktrin?
What positive aspects do you encounter using Doktrin?
What negative aspects do you encounter using Doktrin?
How difficult is it to change between Doktrin and the other systems?
What tasks do you handle using the other systems?
Have you had any problems with the other systems?
Do you get any support to solve the problems that arise from using the systems?
Have you needed to call a patient? Why?
Would you like to change the technical equipment that you use the systems on?
Do you ever work anywhere else than here on 1177 when you are on chat duty?
Would you like to work from home?
Does the patient ever come back and tell you how it went?
(To nurses): Do you ever get any feedback from the doctors about whether your case was correctly categorised?
How much cooperation do you have with the doctors/nurses?
Is it something you would like to change in your digital work environment?

Part 3: Telephone

Now we will focus on when you work on the phone and the systems you use there.

Can you describe what you do when a patient you shall take care of contacts you on the telephone and which systems you use?
Is it the same system you use for the chat? Or are there any others?
What systems do you use most of the time for working on the telephone?
How difficult is it to change between the systems on the telephone?
How do you use the systems?
Do you have any problems using the other systems, the ones you don't use in the chat?
Do you get any support to solve the problems that arise from using all of the systems when you work on the phone?
Do you ever work anywhere else than here on 1177 when you are on the telephone?
Is there anything you would like to change in your digital work environment (unless I have asked before)?
What do you think are the advantages of working with the chat?

For peer review only

What are the advantages of working on the telephone?
Which is more stressful? Why?
Which do you prefer? Why?

Part 4: Conclusion

We will now conclude the interview.

Is there anything you want to add or something you want to ask?

If I may sum up ... (summary of previous answers). Is that right?

Thank you very much for allowing me to interview you - it has been very informative and will help us a lot in our research project.

For peer review only

Standards for Reporting Qualitative Research (SRQR)

Please indicate in which section, figure or table each item has been reported in your manuscript. If you feel that an item does not apply to your manuscript, please enter N/A.

For more information about the SRQR guidelines, please see O'Brien *et al.*, 2014: https://doi.org/10.1097/ACM.0000000000000388

No.	Item	Description	Section
Title and abstract			
1	Title	Concise description of the nature and topic of the study. Identifying the study as qualitative or indicating the approach (e.g., ethnography, grounded theory) or data collection methods (e.g., interview, focus group) is recommended	Title Pg. 1
2	Abstract	Summary of key elements of the study using the abstract format of the intended publication; typically includes background, purpose, methods, results, and conclusions	Abstract Pg. 2
Introduction			
3	Problem formulation	Description and significance of the problem/phenomenon studied; review of relevant theory and empirical work; problem statement	Intro-duction Pg. 3 - 4
4	Purpose or research question	Purpose of the study and specific objectives or questions	Intro-duction Pg. 4
Methods			
5	Qualitative approach and research paradigm	Qualitative approach (e.g., ethnography, grounded theory, case study, phenomenology, narrative research) and guiding theory if appropriate; identifying the research paradigm (e.g., postpositivist, constructivist/ interpretivist) is also recommended; rationale*	2.2 The Research study Pg. 5
6	Researcher characteristics and reflexivity	Researchers' characteristics that may influence the research, including personal attributes, qualifications/experience, relationship with participants, assumptions, and/or presuppositions; potential or actual interaction between researchers' characteristics and the research questions, approach, methods, results, and/or transferability	Contri-butorship statement Pg. 9
7	Context	Setting/site and salient contextual factors; rationale*	2.1 The Case Pg. 4
8	Sampling strategy	How and why research participants, documents, or events were selected; criteria for deciding when no further sampling was necessary (e.g., sampling saturation); rationale*	2.2 The Research Study Pg. 5

9	Ethical issues pertaining to human subjects	Documentation of approval by an appropriate ethics review board and participant consent, or explanation for lack thereof; other confidentiality and data security issues	Ethics statement Pg. 9
10	Data collection methods	Types of data collected; details of data collection procedures including (as appropriate) start and stop dates of data collection and analysis, iterative process, triangulation of sources/methods, and modification of procedures in response to evolving study findings; rationale*	2.2 The Research Study Pg. 5
11	Data collection instruments and technologies	Description of instruments (e.g., interview guides, questionnaires) and devices (e.g., audio recorders) used for data collection; if/how the instrument(s) changed over the course of the study	2.2 The Research Study Pg. 5
12	Units of study	Number and relevant characteristics of participants, documents, or events included in the study; level of participation (could be reported in results)	2.2 The Research Study Pg. 5
13	Data processing	Methods for processing data prior to and during analysis, including transcription, data entry, data management and security, verification of data integrity, data coding, and anonymization/deidentification of excerpts	2.2 The Research Study Pg. 5

14	Data analysis	Process by which inferences, themes, etc., were identified and developed, including the researchers involved in data analysis; usually references a specific paradigm or approach; rationale*	2.2 The Research Study. Pg. 5 The theory described in the Intro-duction section, pg. 3-4
15	Techniques to enhance trustworthiness	Techniques to enhance trustworthiness and credibility of data analysis (e.g., member checking, audit trail, triangulation); rationale*	Contributo rship statement - pg. 9; 2.2 The Research Study - pg. 5.
Results/findings			
16	Synthesis and interpretation	Main findings (e.g., interpretations, inferences, and themes); might include development of a theory or model, or integration with prior research or theory	3. Results Pg. 5
17	Links to empirical data	Evidence (e.g., quotes, field notes, text excerpts, photographs) to substantiate analytic findings	3. Results Pg. 5
Discussion			

18	Integration with prior work, implications, transferability, and contribution(s) to the field	Short summary of main findings; explanation of how findings and conclusions connect to, support, elaborate on, or challenge conclusions of earlier scholarship; discussion of scope of application/ generalizability; identification of unique contribution(s) to scholarship in a discipline or field	4. Discussion Pg. 8
19	Limitations	Trustworthiness and limitations of findings	4. Discussion - pg. 8; Strengths and limitations of the study - pg. 2
Other			
20	Conflicts of interest	Potential sources of influence or perceived influence on study conduct and conclusions; how these were managed	N/A
21	Funding	Sources of funding and other support; role of funders in data collection, interpretation, and reporting	Funding statement - pg. 9

*The rationale should briefly discuss the justification for choosing that theory, approach, method, or technique rather than other options available, the assumptions and limitations implicit in those choices, and how those choices influence study conclusions and transferability. As appropriate, the rationale for several items might be discussed together.

When submitting your manuscript via the online submission form, please upload the completed checklist as a Figure/supplementary file.

If you would like this checklist to be included alongside your article, we ask that you upload the completed checklist to an online repository and include the guideline type, name of the repository, DOI and license in the *Data availability* section of your manuscript.

Developed from: Bridget C. O'Brien, Ilene B. Harris, Thomas J. Beckman, Darcy A. Reed, David A. Cook. Standards for Reporting Qualitative Research: A Synthesis of Recommendations. Academic Medicine, Volume 89, Issue 9, September 2014, Pages 1245-1251. https://doi.org/10.1097/ACM.0000000000000388
